# Hughes’s Reverspectives: Radical Uses of Linear Perspective on Non-Coplanar Surfaces

**DOI:** 10.3390/vision3040063

**Published:** 2019-11-18

**Authors:** Thomas V. Papathomas, Patrick Hughes

**Affiliations:** 1 Laboratory of Vision Research and Center for Cognitive Science, Rutgers University, 152 Frelinghuysen Road, Piscataway, NJ 08854-8020, USA; 2 Department of Biomedical Engineering, Rutgers University, New Brunswick, NJ 08854, USA; 3 Reverspective, 72 Great Eastern Street, London EC2A 3JL, UK; info@reverspective.com

**Keywords:** reverse perspective, forced perspective, depth inversion, illusory motion

## Abstract

Two major uses of linear perspective are in planar paintings—the flat canvas is incongruent with the painted 3-D scene—and in forced perspectives, such as theater stages that are concave truncated pyramids, where the physical geometry and the depicted scene are congruent. Patrick Hughes pioneered a third major art form, the reverse perspective, where the depicted scene opposes the physical geometry. Reverse perspectives comprise solid forms composed of multiple planar surfaces (truncated pyramids and prisms) jutting toward the viewer, thus forming concave spaces between the solids. The solids are painted in reverse perspective: as an example, the left and right trapezoids of a truncated pyramid are painted as rows of houses; the bottom trapezoid is painted as the road between them and the top forms the sky. This elicits the percept of a street receding away, even though it physically juts toward the viewer. Under this illusion, the concave void spaces between the solids are transformed into convex volumes. This depth inversion creates a concomitant motion illusion: when a viewer moves in front of the art piece, the scene appears to move vividly. Two additional contributions by the artist are discussed, in which he combines reverse-perspective parts with forced and planar-perspective parts on the same art piece. The effect is spectacular, creating objects on the same planar surface that move in different directions, thus “breaking” the surface apart, demonstrating the superiority of objects over surfaces. We conclude with a discussion on the value of these art pieces in vision science.

## 1. Introduction

There are several cues that elicit the percept of depth in general [1,2] and from paintings on flat painted surfaces in particular [3,4]. The most important cues are linear perspective [5,6,7], shading [8], occlusion [9], aerial perspective [10], relative size [11,12], foreshortening [13], blur [14,15], binocular disparity [1,15] and texture gradient [16]. Among them, linear perspective, properly used, is the most potent depth cue and it has been used for centuries to enhance the appearance of vivid depth in paintings. The aim of this paper is not to provide a history or a review for the use of linear perspective (the interested reader can consult Kubovy [17], Kemp [18] and Sinisgalli [19] for excellent reviews). Instead, we will consider its use in three types of surfaces that give rise to three distinct classes of perspective art. The first two classes of perspective art have been used widely for a long time. We will concentrate on the third class, which was invented and pioneered by one of the authors (Hughes) in the last 55 years [20,21,22]. 

Let us first consider the most widely known class: Linear perspective on flat surfaces, which we will refer to as *planar perspective*. In its simplest formulation, the idea is to consider a flat transparent painting plate that is frontoparallel to the painter. Lines of sight originate from all points of the scenery behind it and terminate on the painter’s eye, the focal point. These lines intersect the transparent plate at virtual points. All the painter must do is to match the color of the real-world point that originated the line of sight and paint its corresponding virtual point on the plate with that color. Theoretically, this process, when repeated for each point of the scenery, produces an image on the plate that is an exact replica of the scenery. Specifically, the pattern of retinal activation produced by the painted plate is the same as that produced by the scene itself (parenthetically, this ray-tracing method is used in computer graphics algorithms [23]). This process also gives rise to the rules of linear perspective, first formulated by Brunelleschi [18,24,25,26,27,28,29,30,31,32,33,34,35,36] and perfected by painters over the years. It is remarkable that when one views the perspective painting from a vantage point that is different than the focal point used by the artist, one gets a vivid sense of depth, despite geometric distortions of the depicted scene [4,6,7,37,38,39]. It must be noted that: (a) The geometry of the depicted three-dimensional (3-D) scene is incongruent with the planar geometry of the painted surface. (b) Specifically, a physical non-frontoparallel rectangle is transformed into a trapezoid onto the pictorial plane with its perceived further edge being shorter than its perceived near edge, even though they are physically roughly equidistant from the viewer, since they are both lying on the frontoparallel plane of the painting. (c) An important property of linear perspective paintings that are adequately large to map onto a large retinal extent is that they appear to move, ever so slightly, when viewers move past them as they continue to gaze at them [40]. 

The second class is that of *forced (accelerated) perspectives*. The most familiar usage of this class is in theater stage design. The simplest example is a stage that is designed to produce the percept of a regular orthogonal room. The stage designer uses a truncated pyramid in which the far wall is smaller than the front opening of the stage. As a result, the left and right walls are slanted trapezoids, as is the ceiling (in some stages, the floor is also a trapezoid, sloping downward toward the audience). This arrangement produces the impression that the room is deeper than it physically is. Forced perspective can also be used with smaller-scale scenes, such as doll houses, dioramas, etc. The properties of forced perspectives that are in correspondence with the above notes for planar perspectives are: (a) The geometry of the scene (i.e., a truncated pyramid that recedes away from the viewer) is congruent with the painted scenery. (b) Specifically, a physical non-frontoparallel rectangle is transformed into a slanted trapezoid both in the physical structure as well as onto the pictorial plane; in fact, the perceived rectangle’s near edge is physically nearer and larger than its perceived further edge. (c) Typically, when viewers gaze at a forced perspective as they move past it, the scenery appears stationary.

This was the state of affairs until Patrick Hughes entered the picture. This paper provides an overview of three major contributions that he has made in art by introducing innovative uses of linear perspective. His work has attracted the attention of several vision scientists in the last 30 years. We refer to the findings of certain papers that are relevant to our discussion of his three major contributions. However, limited space does not allow us to provide an extensive review of this literature. 

## 2. First Major Contribution: Reverspectives

**Figure 1:**
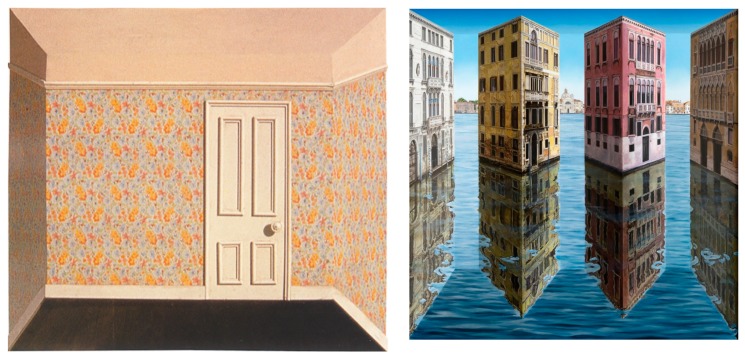
Left panel: “Sticking-out Room” (1964). It must be pointed that this “room” scene is painted on a 3D truncated pyramid that juts toward the viewer. Specifically, what appears to be the far wall with a door is physically closer to the viewer than the outer edges of the floor, walls, and ceiling. Right panel: “Real Reflections” (2017).

Patrick Hughes started a new form of perspective-based art. He started with a very simple example in 1964, “Sticking-out Room”, shown in the left panel of Figure 1. This is a 3-D *convex* truncated pyramid that juts out toward the viewer. What makes it unique is that it is painted realistically as a room, suggesting a *concave* space; the geometry of the surfaces provide some form of perspective, which he called “*reverspective*”, short for “reverse perspective”. In this sense, this piece is the opposite of forced perspective, where both the geometry and the painted scene provide visual cues to perceive a concave space. This simple, yet ingenuous, manipulation was the beginning of a long series of such “contradictory” pieces of art by Patrick Hughes—and his imitators. Reverspectives, as exemplified by “Sticking-out Room”, are bistable stimuli: at close distances, especially when viewed binocularly, they give rise to the veridical percept of a convex surface; at longer distances, especially under monocular viewing, they give rise to the illusory percept and appear to be concave. At intermediate distances, the percept oscillates between the veridical and the illusory state. Here are the main properties of reverspectives: (a) The physical geometry of the art piece is opposite to that of the painted scenery. (b) A physical non-frontoparallel rectangle, such as the left wall in “Sticking-out Room”, is transformed into a slanted trapezoid; unlike in forced perspectives, however, when we are in the illusory state, the perceived (left wall) rectangle’s near edge (the left edge of the wall) is physically further and larger than its perceived further edge. (c) When viewers gaze at a reverspective and obtain a stable illusion, the scenery appears to move vividly as they move past it (illusory motion) [5,20,24].

The simplistic “Sticking-out Room” was the first attempt at a reverspective and it produced a very weak depth-inversion illusion and illusory motion. However, this bold step pioneered a new technique that lay dormant for years, while Patrick Hughes was busy with other forms of art. He went back to reverspective work circa 1990 with “Up the Line and Down the Road” (1991) and, since then, he has been producing much more complex and fascinating art pieces. Photographs of some of these pieces are included here as figures. For example, the right panel of Figure 1 is one of his latest pieces, “Real Reflections” (2017), that shows the tremendous progress in the evolution of reverspective art. It is composed of three vertically elongated truncated pyramids, just as the single one of “Sticking-out Room”, that jut toward the viewer. In addition to its pictorial realism, “Real Reflections” produces a much more compelling depth inversion and very vivid illusory motion than “Sticking-out Room” to a viewer who moves in front of it. We provide some possible explanations for this superiority in the next paragraph. To illustrate the most common elements for the geometry of reverspectives, we provide orthographic front, side and top views of a recent piece, “Making Space” (2016) Figure 2 that consists of four truncated pyramids. In the top and side views the thick arrows indicate the direction of the viewer’s position; this makes it obvious that the truncated pyramids are protruding toward the viewer. Figure 3 is another reverspective, “Stairs to the Stars” (2016), that clearly illustrates how its three truncated pyramids jut toward the viewer.

According to Patrick Hughes, reverspectives evoke illusory motion because they use proprioception to confound the viewer. For instance, if the viewer squats down in front of a scene that shows the interior of a building, one’s view of the reverspective alters in the opposite direction, upwards: specifically, rather than seeing more of the ceiling and less of the floor, as expected, they actually obtain the opposite effect: i.e., they see less of the ceiling and more of the floor, as if they were lifted upward whereas in fact they squatted down. This happens until one’s line of sight lies on the plane that defines the ceiling. Since the information from one’s limbs contradicts one’s eyes, one chooses to disregard this conflict by presuming and assuming that the artwork is moving rather than oneself. The same is true as one moves past the reverspective left to right; it is as if one is moving right to left regarding the planes as perceived by the eyes.

**Figure 2:**
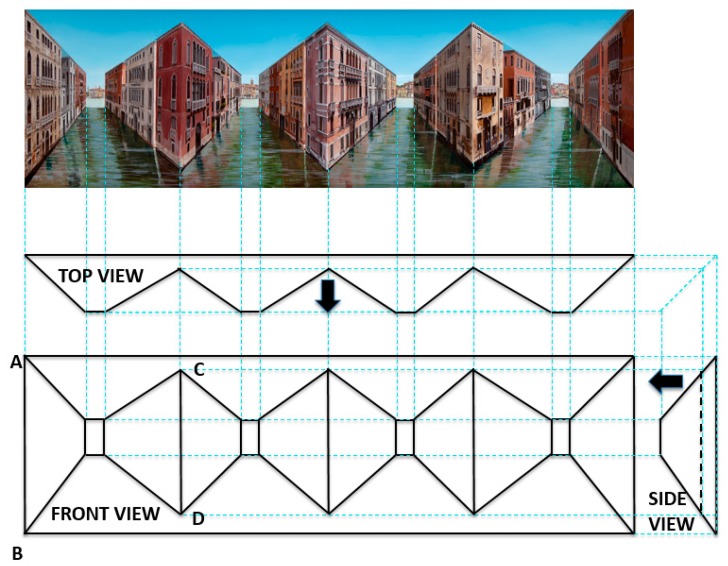
“Making Space” (2016). **Top panel**: Front view of painted piece. **Middle panel**: Top view. Bottom left panel: Unpainted front view. Bottom right panel: Side view. Thick arrows point in the direction of the viewer. See text for details.

**Figure 3:**
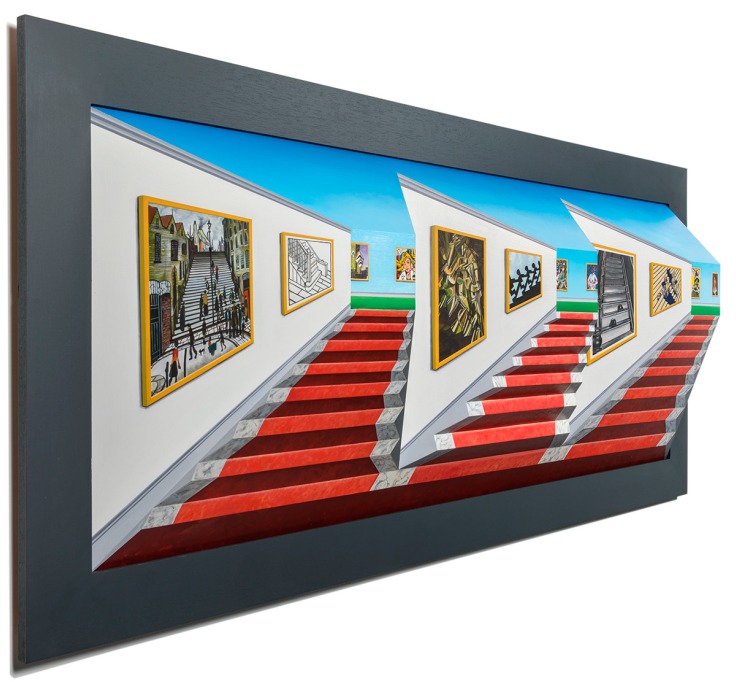
“Stairs to the Stars” (2016).

**Figure 4:**
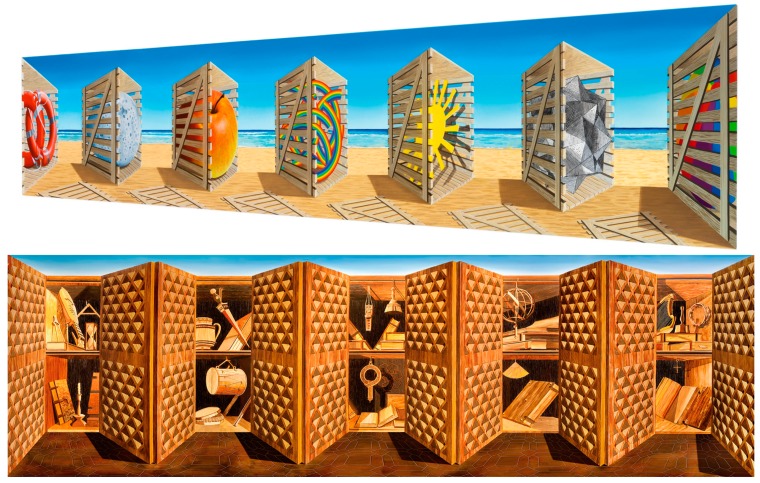
Top panel: “Crate Expectations” (2018). Notice that the central edge of each crate appears to be closer to the viewer than any other part of that crate; in reality, this central edge is the farthest part of the crate. Bottom panel: “A Study of the Studiolo” (2013). The five opened closets appear to be further than the doors, whereas they are closer to the viewer than the doors.

There are three important factors that make pieces such as those in the right panel of Figure 1 and in Figure 2 to produce a much stronger depth inversion and sense of motion than “Sticking-Out Room”:

1) *The presence of “floating” vertical edges*: In addition to the “external” vertical edges that coincide with the physical boundary of the piece, such as edge AB in Figure 2 (as well as the two boundary vertical edges in “Sticking-out Room”), there are also internal floating vertical edges in Figure 2, such as edge CD (no such edges exist in “Sticking-out Room”). External edges are hard to perceive as moving, because they are anchored to physical boundaries. By contrast, internal floating vertical edges appear to move vividly when viewers obtain the depth-inversion illusion. 

2) *The presence of concavities*: The complex reverspectives of Figure 2 and the right panel of Figure 1 contain both concavities and convexities, unlike the simple piece of the left panel of Figure 1 that is purely convex. Concave spaces are more likely to produce depth inversion—and the concomitant paradoxical motion—because humans have a bias to perceive objects as convex [25,26,27]. This bias favors perceiving the convex “Sticking-out Room” as convex, thus weakening the depth-inversion illusion. By contrast, the concave parts of the complex reverspectives tend to be perceived as convex, thus strengthening the illusion. 

3) *Assigning the role of “figure” to concave parts*: This is evident in both the complex pieces of Figure 1 and Figure 2. Specifically from a pictorial point of view, the Venetian buildings form the “figure” against the “ground” of the surrounding water and the sky. It so happens—or is deliberately designed by the second author—that these buildings are painted on concave surfaces. Since viewers’ attention is drawn to the figure and since these figures are concave, the convexity bias acts to invert the perceived depth and strengthen the illusion, due to the superiority of figure parts over shapeless ground. This last factor is powerfully present in “Crate Expectations” (2018) in Figure 4, where the crates stand out as figures but not as potent in “A Study of the Studiolo” (2013) in the same figure, where the doors have a weaker role as figures, because they are juxtaposed with equally elaborate pictorial surroundings. 

**Figure 5:**
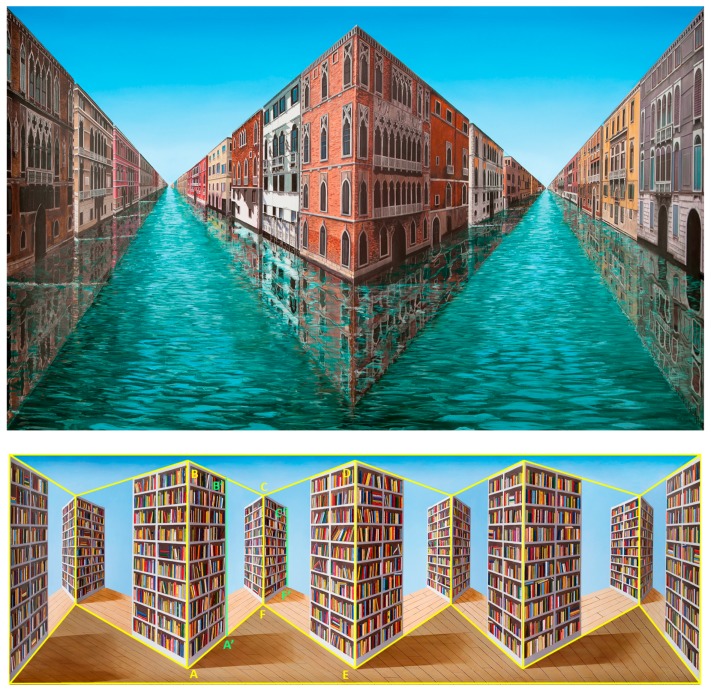
Top panel: “Double Infinity” (2013). Bottom panel: “Reverspective versus Perspective” (2007). See text for details.

In addition to truncated pyramids, Patrick Hughes uses other solid shapes to form his non-planar painting surfaces. Figure 5 illustrates two common geometries. For example, “Double Infinity” (2013) is composed of two pyramids, which allow the painted scene to extend all the way out to the vanishing point, whereas “Reverspective versus Perspective” (2007) uses four vertically oriented “truncated” prisms. More on this piece follows in the next section.

The invention of the reverspective technique was Patrick Hughes most impressive contribution to the use of perspective in three-dimensional paintings. The next two sections present additional contributions that combine reverspective with the other major forms of linear perspective. It must be emphasized that in this paper we will not cover Hughes’s artistic innovations; rather, we cover aspects of Hughes’s contributions that deal with perceptual issues, focusing particularly on the perception of three-dimensional space and the role of linear perspective as a powerful cue to depth. 

## 3. Second Major Contribution: Combining Forced and Reverse Perspective

**Figure 6:**
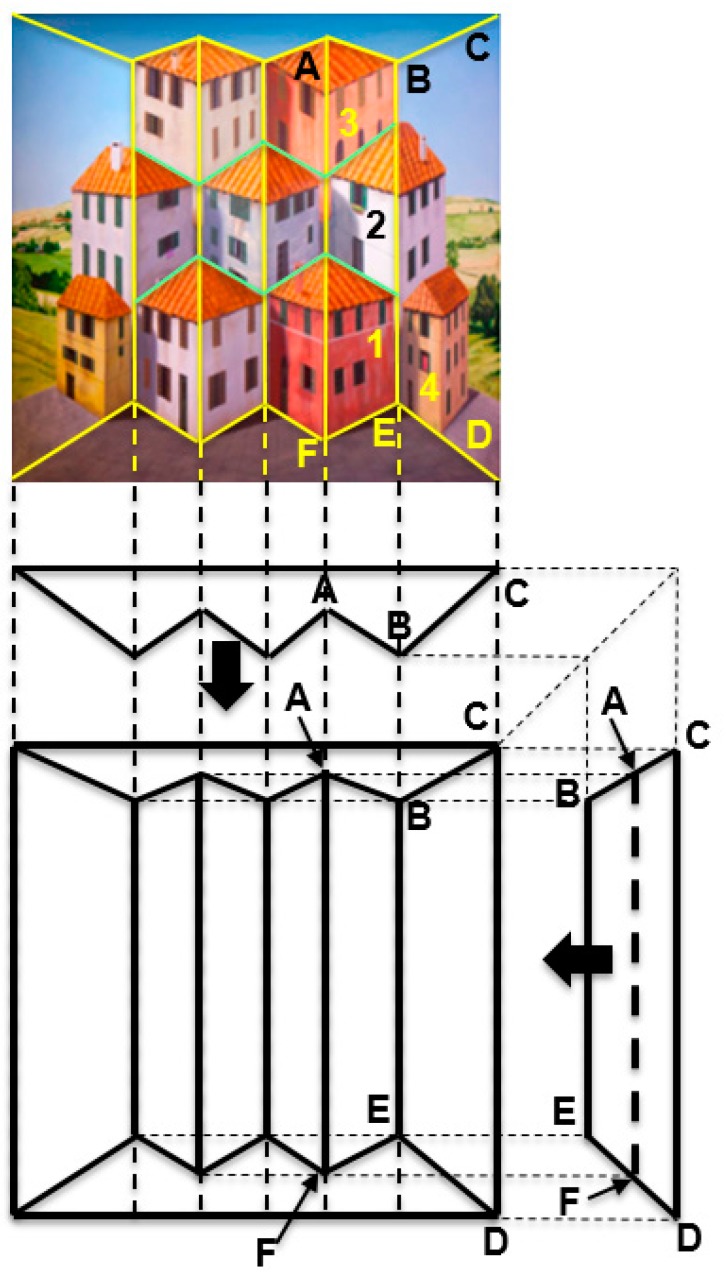
“Poetry in Motion” (2012). See text for details.

Patrick Hughes has a good understanding of how to paint surfaces to yield astonishing effects of transforming the perceived three-dimensional space. In the last decade, he has invented a technique for combining forced and reverse perspective on the same non-planar “canvas”. A typical example is provided by his “Reverspective versus Perspective” (2007) on the bottom panel of Figure 5. Yellow lines have been superimposed on the photograph to indicate the physical edges at the intersection of the planar surfaces that comprise the art piece (more details on the geometry of this piece are provided for a very similar piece in Figure 6). These surfaces form four “truncated” prisms. One of these is labeled as ABCDEF. One can think of this solid being formed by a vertical prism extending vertically to infinity but “truncated” by the intersecting oblique planes of the floor and the ceiling. Please note that the short edge CF of the prism is closer to the viewer than its long edges AB and DE. For purposes of explaining the properties of this piece, we have also used light green lines to indicate vertical painted—not physical edges.

Based on the above notation, it is easy to see that the artist has created both forced-perspective bookcases in the background and reverse-perspective bookcases in the foreground. For example, the near edge CF of the background bookcase is longer than its far edge C’F’, thus creating a congruent relationship between the physical geometry and the painted perspective. On the contrary, the far corner edge AB of the foreground bookcase is longer than its near edge A’B’, thus creating a rivalrous relationship between the physical geometry and the painted perspective. Viewers perceive all bookcases as convex objects with their long edges being closer to them than their short edges. However, under the illusion, the foreground bookcases appear to rotate if the viewer moves in front of the painting, whereas the background bookcases remain stationary. The effect is stunning: one part of a planar surface (such as part ABB’A’ of plane ABCF) appears to be rotating, whereas another part (CFF’C’) appears to be still! The clever painting “tears” the planar surface apart. This is one of the best examples of the superiority of objects (in this case, bookcases) over surfaces. Patrick Hughes has created several other pieces in the genre, such as “The History of Sculpture” (2008) and “Off on a Tangent” (2018).

## 4. Third Major Contribution: Combining All Three Types of Perspective

In his quest for innovative ideas in perspective art, Patrick Hughes has created several 3-D pieces that combine all three major types of perspective, namely forced, reverse, and planar, all on the same piece. The technique in the previous section was based on creating two types of objects, as shown on the bottom panel of Figure 5: (a) Reverse-perspective objects are placed in the background by centering them around far edges, which are long and define a concave dihedral angle; and (b) Forced-perspective objects are placed in the foreground by centering them around near edges, which are short and define a convex angle. Thus, these two classes of objects are placed at very different distances from the observer.

By contrast, Patrick Hughes has invented another technique that places both forced- and reverse-perspective objects at the same distance from the observer. Impressively, this technique allows the third class of planar-perspective objects to also be embedded in the same art piece. A concrete example of this technique is shown by “Poetry in Motion” (2012) in Figure 6. The top panel shows the front view of the painted piece. The three panels at the bottom show orthographic views to illustrate the geometry. Yellow lines have been superimposed on the top panel to indicate physical edges. Light green lines indicate painted—not physical—edges that denote house boundaries. These lines, together with the orthographic views, make it clear that the piece is composed of three “truncated prisms”, such as the one labeled ABCDEF, with their flat faces lying in the back and their sharp edge jutting toward the viewer. 

The originality of this technique lies in the fact that the painted edges—shown by light green lines—separate the houses into three distinct rows of houses with different perspective properties, as follows: The bottom and top rows contain houses, such as those marked as “1” and “3”, that are painted on concave surfaces (the two extreme houses of the bottom row are exceptions: they are planar and are described separately below); the linear perspective lines of these houses, combined with the convexity bias, provide strong cues for depth inversion and the concomitant illusory motion. By contrast, the middle row’s houses are rendered on convex surfaces; this, combined with the linear perspective lines, provide strong cues to perceive them in veridical depth, hence stationary. Finally, the extreme left and extreme right (labeled “4”) houses of the bottom row are painted on a slanted planar surface. Thus, this piece combines all three basic forms of perspective. Importantly, notice that all three types of houses are equidistant from the viewer. As an example, the walls labeled 1, 2, and 3 of the corresponding buildings are all on the same plane, equidistant from the viewer. Other examples of this genre of paintings include “Forced into Reverse Perspective” (2008), “Daydreaming” (2008), “Book Mountain” (2013), “Warholism” (2012) and “Handy” (2012).

The perceptual effects of these arrangements are equally stunning as those in the previous section. Specifically, a moving viewer perceives conflicting unexplainable motions of the various buildings. For example, house #2 appears stationary, whereas #1 and #3 appear to rotate vividly. House #4 appears to rotate ever so slightly. Its motion appears stronger than would have been if this had been the only painted house, all else on the piece left blank (unpainted); perhaps its perceived motion is aided by the vivid motion of the adjacent house #1 of the bottom row. As in “Reverspective versus Perspective” (2007) (Figure 5), there are different motions for different houses. Some of them rotate with the moving viewer, such as houses #1 and #3, whereas others, such as #2, stay fixed. This creates a torsional-like motion between objects that actually belong to the same planar surface; in our example, one of the walls of houses #1, #2 and #3 is painted on the ABEF plane. Yet, these walls move in opposite directions, causing the planar surface ABEF to be “torn” along the edges of the houses, since the top and bottom houses move in a direction different than that of the middle house. Once more, this provides a prime example for the superiority of objects over surfaces. After all, our visual system parses the world into objects that take precedence over surfaces.

## 5. Discussion

Patrick Hughes has produced three main types of 3-D illusions, as described in the last three sections and illustrated in the figures of this paper. They deserve to be included in all compilations of classic illusions because of three main properties: (a) Their effect is impressive, as they transform concave, void, spaces into convex objects, as well as they transform convex volumes into void, hollow spaces by virtue of the scenes that are painted on them. (b) They can be obtained from a wide range of vantage points and viewing distances, even under binocular viewing, unlike some classic illusions such as the Ames Room [28,29] and the Ames Chair [30,31,32], which require that they be viewed only monocularly and from a single vantage point (usually requiring a peephole for proper viewing). (c) They generate vivid illusory motion for viewers who move in front of them. It must be noted for the art pieces described in Section 1, namely the pieces that contain only reverse-perspective cues, the planar surfaces that make up the piece appear to move coherently: each planar surface maintains its flatness and appears to move as a single plane. This is not necessarily the case for the pieces of Section 3 and Section 4, as explained below. 

In addition to the three properties above, the two classes of art pieces described in Section 3 and Section 4 are characterized by the additional property of breaking planar surfaces into objects that move with different 3-D trajectories. Thus, different parts of the same planar surface are painted with conflicting perspective cues such that some parts appear to belong to a convex object while other parts appear to be lying inside of concave spaces. The end result is that these parts appear to move in different directions, which is a physical impossibility, since they are parts of the same planar surface. This fragmentation of surfaces into objects affects the appearance of lines in 3-D space. For example, line AF in Figure 6 is a single vertical straight line, as shown in the front and side orthographic views of “Poetry in Motion” (2007). However, when a viewer obtains the depth-inverting illusion, this straight line is fragmented into two non-collinear line segments: the bottom line segment (defining the middle corner of Building 1), appears to be closer than the top segment (separating Building 2 from the building to its left and extending collinearly to become the middle corner of Building 3). Thus, a straight line is broken into two segments that are at different distances from the observer!

It must be noted that the illusory motion is perceived only when an art piece elicits the percept of depth inversion. Specifically, when moving observers perceive the illusion of inverted depth, they also perceive the entire scenery to move vigorously, and vice versa: when they perceive illusory motion, they also perceive the illusory inverted depth. By contrast, when they obtain the veridical 3-D geometry of the art piece, the scenery appears stationary for moving viewers. This equivalence between depth inversion and illusory motion can be used to verify the veracity of viewers’ reports that they have obtained depth inversion while they view a piece as they stand motionless in front of it. In some experiments, we need to confirm that they obtain depth inversion *perceptually* (namely as a result of processing within the visual system), rather than only *conceptually* (namely as a result of cognitively visualizing the depth-inverted scene without actually perceiving it as such). In the first case, the piece appears to move when they move whereas, in the second case, they do not experience illusory motion. Thus, to ensure that they experience a perceptual depth inversion when they report that they achieve depth inversion, the experimenter can ask them to move back and forth and report their percept. If they report that they perceive the piece appears to move as they move, this serves as confirmation that they have indeed obtained a stable perceptual depth inversion. If they report that the piece is stationary, there are two distinct possibilities: either they never achieved depth inversion or, even if they had achieved it while they were stationary, the illusion broke down as they started to move because the motion parallax cue provided information that broke the illusion and revealed the veridical geometry.

The focus of this paper is on key contributions by Patrick Hughes in reverse-perspective art and its combinations with forced and planar perspective. Because of space constraints, we conclude the paper with a very brief discussion on its value and potential in vision research. This is by no means meant as a thorough review of the relevant literature, which requires a separate review altogether.

### 5.1. The Influence of Prior Experiences on Visual Perception

The interaction of depth cues in perceiving reverspectives is a key question in this area. Thus far, the main cues that have been studied are binocular disparity, linear perspective and motion parallax [5,33,34]. It appears that prior experiences, specifically with retinal perspective projection, play a leading role in the depth-inversion illusion. Linear perspective cues overcome the influence of data-driven bottom-up cues, such as binocular disparity and motion parallax. In this regard, this is very similar to the case of the hollow-mask illusion, where prior knowledge of faces being convex also dominates over data-driven cues to elicit depth inversion [35]. Brain imaging can offer insight on how data-driven signals and stored knowledge interact in perceiving depth-inversion illusions in general and reverspectives in particular [36,41].

### 5.2. Clinical Applications

Starting with Emrich in 1989 [12], it has been observed that schizophrenia patients do not perceive depth-inversion illusions as strongly as healthy controls. One of the most plausible hypotheses is that the influence of prior experience in patients is much weaker than in controls [41]. Much of the research in this area has been conducted using the hollow-mask illusion [42]. Extending this type of research to reverspectives removes the potential confounding influence of affective factors with hollow faces, especially in patients.

### 5.3. Perception Under Self-Motion–Visuo-Motor Response

As a rule, when humans move in stable stationary surroundings, they have no problem perceiving this stable environment and navigating in it based on visual, vestibular, and proprioceptive signals. Reverspectives appear to defy this rule because, even though they are stationary, they appear to move vividly for moving observers. Thus, they offer suitable stimuli for testing visual perception under self-motion [43]. In addition, because their perceived illusory space is drastically different than the veridical space, they can be used to address the important issue of interactions between the dorsal and ventral visual pathways; they can be used as stimuli in experiments that study whether responses are governed by the perceived, rather than the veridical, stimulus [44].
